# ASSOCIATION OF MULTIMORBIDITY WITH INCIDENT SHINGLES AMONG OLDER AMERICANS

**DOI:** 10.1093/geroni/igz038.972

**Published:** 2019-11-08

**Authors:** Hyewon Kang, Eileen Crimmins, Jennifer A Ailshire

**Affiliations:** 1 University of Southern California, Los Angeles, California, United States; 2 Davis School of Gerontology, University of Southern California, Los Angeles, California, United States

## Abstract

Older adults with multimorbidity are susceptible to shingles due to deterioration of immune function related to coexisting chronic disorders and immunological change. Although the association between individual chronic diseases and incident shingles has been documented, little is known about the effect of having multiple conditions on the risk for developing a new case of the disease. Multimorbidity is a normal condition with advanced aging. This paper examines risk of shingles onset associated with multimorbidity defined as having one or more chronic diseases including hypertension, diabetes, cancer, heart disease, lung disease, arthritis, and stroke) among older American. Data for this study come from the 1992 -2016 Health and Retirement Study. The study finds that risk for onset of shingles linearly increases with number of chronic disorders when age, gender, and race/ethnicity were adjusted (adjusted OR: 1.58, 95% CI; 1.37, 1.80 for those with 1 chronic condition vs adjusted OR: 3.43, 95% CI: 2.21, 5.53 for those with 6 conditions). The risks for multimorbidity were little changed after additional adjustments for socioeconomic status and health behaviors. The effect of multimorbidity on developing shingles is greater among young-old adults in their 50s than among the older-old adults aged 80 and higher. Our finding challenges the single-disease framework that is often used in previous studies examining risk factors for shingles. Our finding highlights the need for primary prevention and treatment of multimorbidity for reduction of shingles cases among older adults, especially young old adults in 50s.

